# Evaluation of PET Scanner Performance in PET/MR and PET/CT Systems: NEMA Tests

**DOI:** 10.4274/mirt.97659

**Published:** 2018-02-01

**Authors:** Mustafa Demir, Türkay Toklu, Mohammad Abuqbeitah, Hüseyin Çetin, H. Sezer Sezgin, Nami Yeyin, Kerim Sönmezoğlu

**Affiliations:** 1 İstanbul University Cerrahpaşa Faculty of Medicine, Department of Nuclear Medicine, İstanbul, Turkey; 2 Yeditepe University Faculty of Medicine, Department of Nuclear Medicine, İstanbul, Turkey; 3 Epsilon Landauer Company, İstanbul, Turkey

**Keywords:** PET/MR, PET/CT, NEMA tests, quality control

## Abstract

**Objective:**

The aim of the present study was to compare the performance of positron emission tomography (PET) component of PET/computed tomography (CT) with new emerging PET/magnetic resonance (MR) of the same vendor.

**Methods:**

According to National Electrical Manufacturers Association NU2-07, five separate experimental tests were performed to evaluate the performance of PET scanner of General Electric GE company; SIGNATM model PET/MR and GE Discovery 710 model PET/CT. The main investigated aspects were spatial resolution, sensitivity, scatter fraction, count rate performance, image quality, count loss and random events correction accuracy.

**Results:**

The findings of this study demonstrated superior sensitivity (~ 4 folds) of PET scanner in PET/MR compared to PET/CT system. Image quality test exhibited higher contrast in PET/MR (~ 9%) compared with PET/CT. The scatter fraction of PET/MR was 43.4% at noise equivalent count rate (NECR) peak of 218 kcps and the corresponding activity concentration was 17.7 kBq/cc. Whereas the scatter fraction of PET/CT was found as 39.2% at NECR peak of 72 kcps and activity concentration of 24.3 kBq/cc. The percentage error of the random event correction accuracy was 3.4% and 3.1% in PET/MR and PET/CT, respectively.

**Conclusion:**

It was concluded that PET/MR system is about 4 times more sensitive than PET/CT, and the contrast of hot lesions in PET/MR was ~ 9% higher than PET/CT. These outcomes also emphasize the possibility to achieve excellent clinical PET images with low administered dose and/or a short acquisition time in PET/MR.

## Abstract (tr)

**Amaç:**

Bu çalışmada son yıllarda nükleer tıpta rutin uygulamaya giren pozitron emisyon tomografisi/manyetik rezonans (PET/MR) görüntüleme sistemi ile PET/bilgisayarlı tomografi (BT) görüntüleme sistemlerinde ‘National Electrical Manufacturers Association’ (NEMA) testlerinin yapılması ve sonuçların karşılaştırılması amaçlandı.

**Yöntem:**

NEMA NU2-07 testlerinden uzaysal ayırma gücü, hassasiyet, saçılma fraksiyonu kayıp sayımlar ve rastgele olay ölçümleri, görüntü kalitesi, sayım kayıpları rastgele olay düzeltme doğruluğu olmak üzere beş ayrı deneysel çalışma yapıldı.

**Bulgular:**

Sensitivite testi sonuçları değerlendirildiğinde PET/MR sisteminin PET/BT sistemine göre yaklaşık 4 kat daha üstün olduğu bulundu. Görüntü kalitesi test sonuçlarından sıcak lezyon kontrastının PET/MR’da %9 daha yüksek olduğu bulundu. PET/MR scatter oranı gürültü eşdeğeri sayım hızı (NECR) 218 kcts ve aktivite konsantrasyonunun 17,7 kcts/cc olduğu durumda %43,4 bulundu. Buna karşılık PET/BT scatter oranı NECR 72 kcts ve aktivite konsantrasyonunun 24,3 kcts/cc olduğu durumda %39,2 bulundu. Tesadüfi hataları düzeltme doğruluğu PET/MR’da 3,4 ve PET/BT’de 3,1 bulundu.

**Sonuç:**

PET/MR’da sensitivite değerinin PET/BT’ye göre yaklaşık 4 kat daha yüksek olduğu, PET/MR’da sıcak lezyon kontrastının PET/BT’ye göre %9 daha yüksek olduğu ayrıca PET/MR’da PET görüntülerinin daha az aktivite miktarı/veya daha kısa çekim süresinde elde edilebileceği sonucuna varıldı.

## INTRODUCTION

Positron emission tomography/magnetic resonance (PET/MR) has been increasingly considered the cutting edge technology in nuclear medicine. The merger of two different scanner technologies, PET and magnetic resonance imaging (MRI), presented more advanced and reliable means of cancer diagnosis. The advent of innovative labeling molecules promotes this marvel of novelty to be an ideal technology joining best of the nuclear and radiological hybrid imaging modalities for tumor detection and treatment. PET/computed tomography (CT) combination has been used in oncological investigations with long-standing experience and knowledge. Such premium changes in PET scanner design rendered PET/MR technology more versatile and promising evidenced by the enhanced tissue contrast and absence of radiation hazards (1).

Generally, the magnetic field has great influence on the ferromagnetic materials such as iron and nickel. Therefore, the conventionally used metallic photon multiplier tubes (PMTs) in PET/CT are not applicable for PET/MR configuration. Instead, silicon photo multipliers (SiPM) and avalanche photodiodes have been introduced as PET/MR compatible photodetectors (2). Accordingly, one of the most important differences between PET/MR and PET/CT equipment is attributed to the photomultiplier’s structure and properties. Semi-conductor detectors are mostly superior to the normal PMTs in terms of quantum efficiency and signal quality (3). Nowadays, the providers might start to replace the classical PMTs by SiPM in the new generations of PET/CT devices (4).

Attenuation correction of PET/CT images is made by using attenuation coefficients (μ) derived from CT map (5). In contrast, attenuation correction of PET images in PET/MR is accomplished via variable algorithms that are not as precise as CT. MR image-based atlas and segmentation methods are the most common algorithms, where the attenuation coefficients obtained from CT images are no longer available in PET/MR (6).

National Electrical Manufacturers Association (NEMA) tests of PET scanners has been firstly introduced as NU 2-1994 for performance assessment. It was published by Society of Nuclear Medicine working group in 1991. Meanwhile, similar standards were being conducted in Europe. However, NEMA and ICE are different standards dedicated for the same purpose. Currently, NEMA standards have been updated through new version (NU2-07). The new update is so far similar to ICR standards that renewed in 2002 and 2007. NEMA tests are performed and recorded prior to accepting new devices. NEMA criteria substantially provides appropriate methods to carry out the performance tests, but never specifies any reference limit. The manufacturer should undertake informing the installation site about the reference values and whether the device is sensitive to possible faults and changes in the ambient conditions such as temperature, humidity, etc. In case of inconsistence between the reference values and quality control results, calibration of the machine ought to be performed and thereafter repeating the quality control tests to evaluate the performance again (7). Three performance parameters are forming the baseline of image quality including spatial resolution, contrast and noise (8). Moreover, different standard phantoms were developed for performing NEMA tests and acquiring PET images. All required algorithms to explore the image quality are available, and the conformity of the images obtained from the device is compared to the global standards. The reference values are saved to be used in the upcoming quality control tests of the equipment (9).

The aim of this study was to evaluate the PET scanner’s performance of PET/MR and PET/CT systems, which are provided by the same vendor with variation due to different PET scanner design, PMT type, and attenuation correction algorithms.

## MATERIALS AND METHODS

In the current study, NEMA tests (NEMA NU2-07) were performed on PET/CT and PET/MR systems. Both products belong to the same company and supplied with time of flight (TOF) technology: PET/MR General Electric GE Company (SIGNATM model) and PET/CT GE (Discovery 710 model). The properties of the PET scanner of PET/MR system are outlined as axial field of view (FOV) 25 cm, crystal size 4×5.3×25 mm, LYSO scintillator, trans-axial FOV 60 cm and SiPMTs. While these for the PET/CT were designed as: axial FOV 15.7 cm, crystal size 4.7×6.3×25 mm, LYSO scintillator, trans-axial FOV 70 cm and metallic PMTs. the varied technical aspects of PET scanner in both PET/MR and PET/CT might arise the difference on the performance.

An ethical consent was not obtained since the study was performed on body phantoms.

### NEMA Tests (NEMA NU2-07)

### Spatial Resolution

This test set the capability of the PET system to localize the position of a point source after image reconstruction and to measure the tomographic spatial resolution of the device in air (non-scattering medium) with ^18^F radioisotope. The spatial resolution of the PET system represents the potential of separation between two points after reconstruction in three-dimensional views. Point spread function is often used to reflect the spatial resolution after reconstruction by measuring the photo-peak full width at half maximum (FWHM) and the full width at tenth maximum (FWTM) of 10%.

### Measurement Method

**Figure 1:**
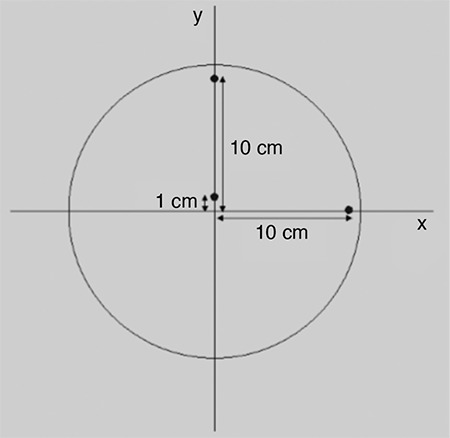
Sources positioning for spatial resolution test

Point sources were obtained from 5 mCi ^18^F solution, then inserted into three capillaries with an inner diameter ≤1 mm and outer diameter ≤2 mm. The sources were placed on the transverse and axial axis at t 1 cm from the center and 10 cm radial offset (Figure 1). 100 000 counts were acquired and filtered back projection was used for multidimensional image reconstruction.

### Sensitivity

**Figure 2:**
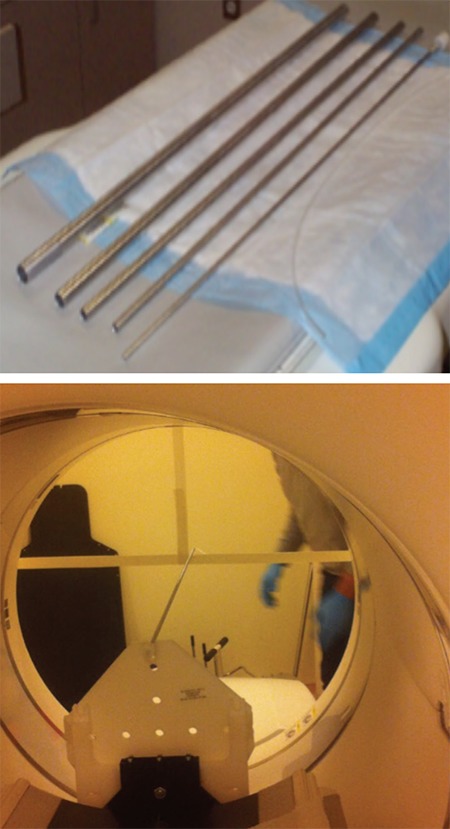
National Electrical Manufacturers Association sensitivity phantom on the (up), centering the source on the (right)

The detector sensitivity of PET device is defined as the count per unit time of the source activity. The purpose of this test is to measure the ability of the scanner system to detect the annihilations resulting from positrons interaction. The measurements were obtained by using specific phantom made up of 5 aluminum sleeves (tubes) that can be inserted in each other. The internal diameters of the bars are between 3.9 mm - 16.6 mm and the external diameters are between 6.4 mm - 19.1 mm. The thickness of the five aluminum sleeves is equal to 1.25 mm and the length is fixed as 700 mm (Figure 2).

### Measurement Method

A low-activity (~ 135 μCi, 5 MBq) of ^18^F was prepared to measure sensitivity values. A portion of the 700±5 mm plastic tube was filled by ^18^F mixed with water, then the tube was closed at the two ends. The phantom was positioned trans-axially at the center of FOV, so that a supporting device was required to keep it outside the imaging field. At least 10000 true counts per slice were acquired. This experiment was repeated in two separate locations, at center and 10 cm away from the center. To report the result, average sensitivity obtained from 5 sleeves was evaluated and extrapolated to find the corresponding sensitivity with no attenuation.

### Scatter Fraction (SF), Count Rate Performance

Annihilation photons emanated from the patient’s body are detected when they hit the PET detectors in a coincidence event. Meanwhile, the events that occur in the detector are classified as random and scatter in addition to the true counts.

The SF of this test measures the sensitivity of the scanner to coincidence events caused by scatter, while the count rate test measures the performance of the PET scanner across variable radioactivity levels. The SF measurement is done at variable activity levels involving negligible system dead time and random events. Scatter was calculated within a radius of 12 cm from center of phantom while scatter photons under the peak was estimated by interpolation between ±2 cm from the center.

### Measurement Method

The test phantom consists of a polyethylenated cylinder with an outer diameter of 203 mm, 700 mm length and 0.96 g/cm3 density. A hole with a diameter of 6.4 mm extends parallel to the central axis of the cylinder at a radial distance of 45 mm. The test phantom was a rod source made of polyethylene or polyethylene coated plastic tube with 800 mm length, the inside diameter was 3.2 mm and the outside diameter was 4.8 mm. This tube was filled with 35 mCi (5.2 mL) ^18^F and placed in the test phantom through the 6.4 mm diameter hole. To start the test, the highly active source is placed in the FOV of the PET device and many measurements were obtained until the activity in the phantom was quite decayed. Owing to these measurements, true (T), scatter (S), random (R) events are separately counted. Then the rate of scattered counts (SR, equation 1) and the noise equivalent count rate (NECR, equation 2) was calculated with the functions shown below:

### Image Quality

**Figure 3:**
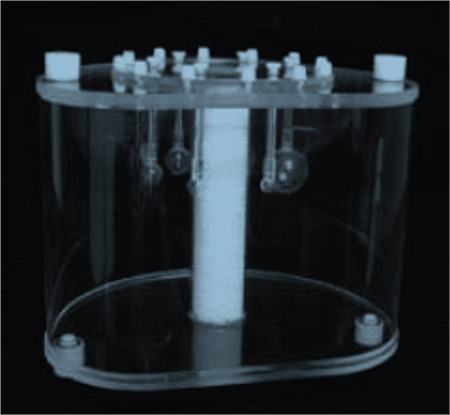
Positron emission tomography body phantom, the middle pipe represents lungs, six fillable spheres with inner diameters of 10, 13, 17, 22, 28 and 37 mm

The aim of this test is to simulate whole body imaging with hot and cold lesions. Body phantom (IQ) was used with different fillable spheres (Figure 3). The contrast was calculated for both hot and cold spheres. The attenuation and scatter correction accuracy was determined from the uniform background and the residual lung activity.

All corrections were made during image reconstruction with similar imaging parameters in terms of image matrix size, pixel size, slice thickness, reconstruction algorithms, filters and other smoothing applications. VUE point FX with 2 iterations / 28 subsets and 5 mm filter cutoff was employed for image reconstruction in PET/MR, and VUE point FX with 3 iterations / 24 subsets and 5.5 mm filter cutoff in PET/CT. Four classes Dixon method was used for photon attenuation correction in PET/MR.

### Image Quality and Calculation Method

Initially, background activity concentration of the body phantom was about 0.14 μCi/cc (±5%). This corresponds to the concentration of typical whole body imaging (10 mCi/70000 cc). Hot lesions were filled with activity ratio 8:1 to that in background while the cold lesions were filled with free water. The phantom with 700 mm linear line was filled with 3.08 mCi ^18^F that is sufficient for activity concentration equal to the background activity concentration of the body phantom. Two large spheres (28 and 37 mm) were filled with water for imitating cold lesions and the other spheres (10, 13, 17 and 22 mm) were filled with ^18^F analogous to hot lesions.

**Figure 4:**
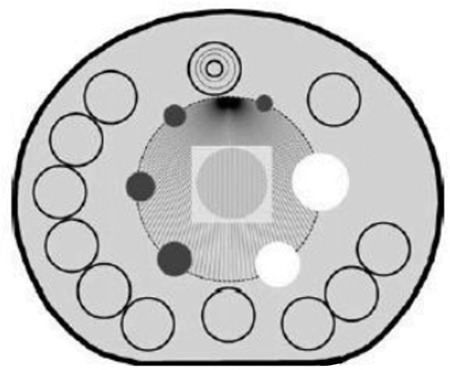
Background regions of interest for image quality analysis

The analysis was made on transverse sections in which a circular regions of interest (ROI) was drawn as close as possible to the dense inner diameter of every cold and hot sphere. For background, ROIs of the same size of the ROIs drawn around the hot and cold spheres were delineated near the edge of phantom up to 12 background ROIs per sphere (Figure 4).

Lesion contrast calculation was performed according to equation (3).

### Count Loss and Random Event Correction Accuracy

The accuracy of count losses and random correction is measured by comparing the true counts rate, where count losses and random correction are made, to the rate derived from measurements with negligible count losses and random events. The data acquired for the count rate and scatter fraction test was also used for this test.

**Figure 5:**
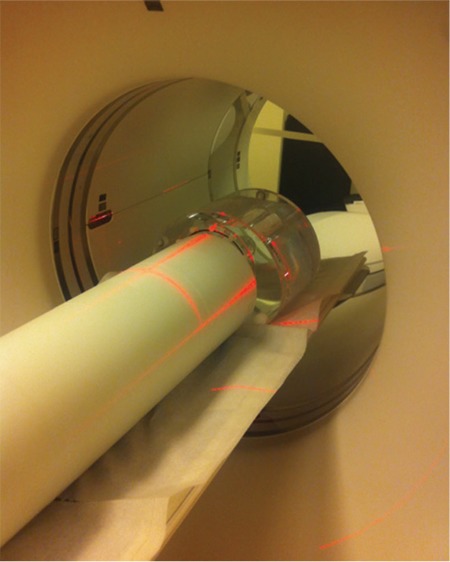
Positioning of polyethylene and body phantom

The line source of the phantom was placed at the closest area to the bed (Figure 5). Body phantom was placed on the tip of the phantom. From the high counting rate to the low rate, subsequent images were acquired with 500,000 counts at certain time intervals. Then, true counts were measured at high and low activity levels. True count rate at low activity levels was extrapolated to determine the amount of deviation (percentage of error) from that at high activity level.

## RESULTS

### Spatial Resolution

The measured spatial resolution of PET/MR and PET/CT were shown in a pattern of FWHM and FWTM on transverse and axial dimensions. The deviations in the localization on the three axes (x, y, z) were given in Table 1. In addition, the measurements obtained at 1 cm and 10 cm radial offset from the center is seen in Table 2.

### Sensitivity

**Figure 6:**
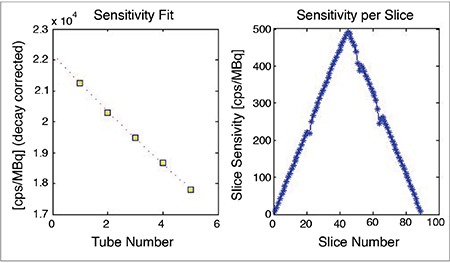
Measurement of sensitivity in positron emission tomography/magnetic resonance. On the left, counts decrease as the rod’s thickness increases due to attenuation. On the right, the measured sensitivity changes per section from outside to inside and from inside to outside

The sensitivity measured at the center of gantry and 10 cm radial offset in PET/MR was 22.2 cps/kBq and 21.74 cps/kBq, respectively. The acceptance limit given by the manufacturer is 21.97 cps/kBq for PET/MR (Figure 6). In comparison to PET/CT, the sensitivity was measured as 5.458 cps/kBq at the center of gantry while the sensitivity at 10 cm from the center was not measured. The limit of acceptance given by the manufacturer is 8.9 cps/kBq.

### Scatter Fraction, Dose Rate Performance

### Scatter Fraction

**Figure 7:**
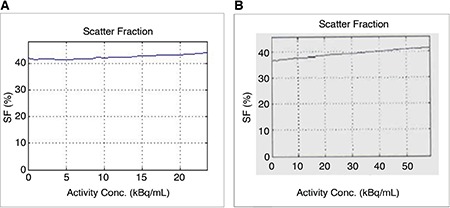
Positron emission tomography/magnetic resonance (A), positron emission tomography/computed tomography (B) measured scatter fraction. SF: Scatter fraction

**Figure 8:**
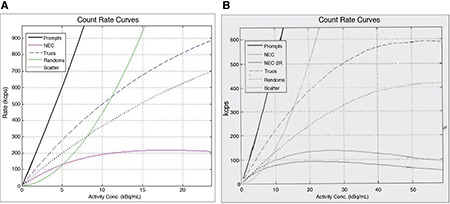
Positron emission tomography/magnetic resonance (A), positron emission tomography/computed tomography (B) measured noise equivalent count rate values

The scatter fraction in PET/MR was 43.4% at NECR peak 218 kcps and corresponding activity concentration 17.7 kBq/cc, in which the scatter fraction limit supplied by the manufacturer was 45%. PET/CT system showed less scatter fraction as 39.2% at 72 kcps NECR peak corresponded to activity concentration of 24.3 kBq/cc, while the reference value provided by the manufacturer was 42% (Figure 7, 8).

### Count Loss measurement

In PET/MR, NECR value was measured as 218 kcps at activity concentration of 17.7 kBq/cc. The limit value of NECR was 210 kcps as ecommended by the manufacturer is given as 210 kcps. NECR value in PET/CT was 72.0 kcps at activity concentration of 24.3 kBq/cc. The manufacturer’s NECR limit value was given as 68 kcps (Figure 8).

### Image Quality

**Figure 9:**
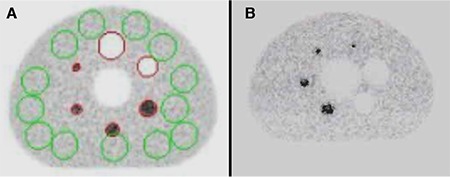
Body phantom image quality. Positron emission tomography/magnetic resonance (A), positron emission tomography/computed tomography (B)

Contrast values of hot spheres with 10, 13, 17, and 22 mm diameter in PET/MR were 56%, 72%, 78% and 85%, respectively, while the provided contrast values from the manufacturer were 30%, 35%, 45% and 55%. Contrast values of background ROIs of the hot spheres were found to be 7.8%, 5.9%, 5.1%, 5.3%, 5.7% and 6%. A threshold value of 10% is given for lung residual activity while the measured value was (1.2%). Contrast value of hot spheres with 10, 13, 17, and 22 mm diameter in PET/CT was found as 53%, 66%, 72% and 79%, respectively. Contrast values provided by the manufacturer are 20%, 30%, 40% and 50%. Contrast values of background ROIs of the hot spheres were 9.5%, 7.6%, 6%, 4.2%, 3.6% and 3%, respectively. A threshold value of 20% is given for the residual activity of lung. The measured value was %13.5 (Figure 9).

### Count Loss and Random Event Correction Accuracy

True counts rate at high activity level were obtained from 81 slices of polyethylene phantom and the changes in the maximum and minimum values of true counts at low activity level were analyzed. The true count rate at low level was extrapolated to determine the amount of deviation (percent error) from that at high activity level. The measured percentage error in PET/MR was 3.4% while it was found 3.1% in PET/CT.

## DISCUSSION

The endeavor of the present work is to evaluate the performance of PET scanner as a part of two different modalities manufactured by the same vendor. The contrast values of 13 and 22 mm hot spheres in PET/MR was calculated to be 72% and 85%, respectively, while the contrast values in PET/CT for 13 and 22 mm hot lesions were 53% and 78%, respectively. The sensitivity at the center was 22.2 cps/kBq in PET/MR, and 5.48 cps/kBq in PET/CT. In comparison, Yester et al. (10) investigated the performance characteristics of GE PET scanner (Company/Discovery 710 model) using NEMA tests. The reported spatial resolution in that study was 4.6 mm at 1 cm from the center, and 5.2 mm at 10 cm. Likewise, the average spatial resolution in our study at 1 cm and 10 cm was 4.8 mm and 5.3, respectively. The sensitivity was also reported as 7.1 cps/kBq at the center and the contrast of hot spheres was reported as 70% and 80% for hot spheres of 13 mm and 22 mm diameter. In general, the sensitivity of PET/MR system seems to be higher owing to the fact that PET detectors are functioning with new SiPMT technology. The assembly of SiPMTs is composed of numerous microcells that result in larger detection efficiency, small physical profile, and supply high gain with low operating voltage (20-80 v).

New features of PET gantry including wider axial FOV (25 cm) and less ring diameter in PET/MR improve the scanner sensitivity and spatial resolution compared with the conventional PET/CT gantry. Similarly, Grant et al. (11) published a study involved implementation of NEMA NU 2-2012 protocol to evaluate PET performance in PET/MR (GE SIGNATM model) including: spatial resolution, NECR, sensitivity, accuracy, and image quality. The scanner showed an average of 4.4, 4.1, and 5.3 mm FWHM for radial, tangential, and axial spatial resolutions, respectively, at 1 cm from the trans-axial FOV center. The peak NECR of 218 kcps and a scatter fraction of 43.6% were achieved at activity concentration of 17.8 kBq/ml. The sensitivity at the center was 23.3 cps/kBq. The maximum relative slice count rate error below peak NECR was 3.3%, and the residual error from attenuation and scatter corrections was 3.6%. The study also mentioned that continuous MR pulsing had either no effect or a minor effect on each measurement.

Additional discrepancy between PET/MR and PET/CT systems originated from the difference in the attenuation correction algorithms (12). The applied attenuation correction in GE Signa PET/MR is atlas based for brain studies and Dixon 4-classes segmentation for the remaining whole body. The MR based attenuation correction of PET/MR is unlike CT based attenuation correction, since it does not directly measure the attenuation coefficients of tissue. Instead, the attenuation is made from information about proton density and relaxation time (fat and water) named by Dixon segmentation. This method has been compared to CT based correction with satisfactory consistence. On the other hand, certain anatomical structures still constitute a challenge to PET/MRI; for example; lung, air, bone and metallic regions like implants. These objects might show quite low MR signal despite their different attenuation properties. However, several studies comparing the efficacy of PET/MR and PET/CT systems in terms of lesion detection stated limitations related to attenuation correction algorithms used in PET/MR. Drzezga et al. (13) performed a study involving twenty-two patients who were subjected to both PET/MR and PET/CT imaging with a standard protocol. The attenuation corrections of PET/MR images were performed using Dixon segmentation and the attenuation correction of PET images was also derived from CT map. As a result, all lesions were detected in PET/MR and PET/CT, and no differences were reported between the two modalities. Wiesmüller et al. (14) reported that 99.2% of the visible lesions in PET/CT were also detected in PET/MR, while 4 patients had extra lesions identified only in PET/MR. There are several experimental trials has been made using NEMA IQ phantom. For instance, Oehmigen et al. (15) evaluated PET/CT and PET/MR images of IQ phantom filled with 8:1 lesion/background ^18^F concentration ratio. It was determined that images in PET/MR can be acquired at the same level of quality as PET/CT with reducing the activity ratio by 3 times owing to the high sensitivity.

In PET/CT, photon attenuation coefficients are usually derived from the patient’s CT images (CT µ map) and PET counts correction is then made pixel by pixel. While in PET/MR where patient’s CT images are no longer available, many studies indicated that the algorithms obtained from MR images could be successfully implemented to correct PET/MR images. One of the most relevant studies on this subject was reported by Martinez-Möller et al. (16). Throughout this study, standard uptake values were evaluated in 35 patients with multiple lungs lesions, bones and neck region. They performed attenuation corrections via CT and MR images of the same patients with data derived from CT (4 classes - segmented attenuation map from CT) and they concluded that there was no significant difference between the algorithms operated in PET/MR as compared to PET/CT results. In fact, there is no similarity between annihilations emission in PET and MR signal to be used in the attenuation correction. In addition, MR- image based and CT-based attenuation algorithms for NEMA IQ phantom were compared in which the image quality and contrast were found to increase with the CT-based attenuation correction (17).

Karlberg et al. (18) compared the results of NEMA tests of PET/CT with TOF and Siemens PET/MR without TOF. It was shown that the sensitivity, NECR values and lesion contrast measurement results were superior in PET/MR when compared to PET/CT. Finally, in the present study, the mean contrast value of five hot lesions in PET/CT was 67.5% while it was 72.7% in PET/MR. The image contrast in PET/MR was superior to PET/CT taking into consideration both systems are incorporating TOF technology. Count loss and random event correction accuracy was also within a close range and acceptable limits.

## CONCLUSION

It was concluded that the sensitivity of PET/MR system is about 4 folds larger than PET/CT, and the contrast of PET/MR was ~ 9% higher than PET/CT, indicating that excellent clinical PET images might be achieved with low administered dose and/or a short acquisition time in PET/MRI acquisition.

## Figures and Tables

**Table 1:**
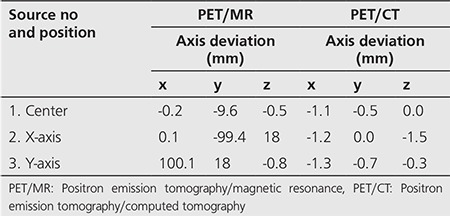
Deviations in the position of the sources for spatial resolution measurement in positron emission tomography/magnetic resonance and positron emission tomography/computed tomography equipment

**Table 2:**
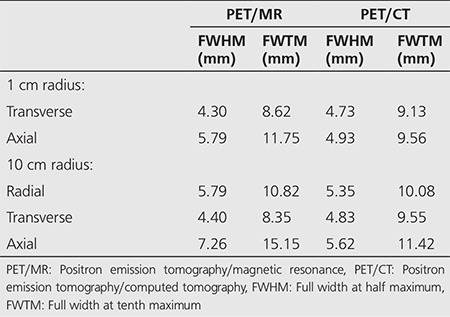
Spatial resolution values in positron emission tomography/magnetic resonance and positron emission tomography/computed tomography
